# Epidermal growth factor-like domain protein 6 recombinant protein facilitates osteogenic differentiation in adipose stem cells via bone morphogenetic protein 2/recombinant mothers against decapentaplegic homolog 4 signaling pathway

**DOI:** 10.1080/21655979.2022.2037380

**Published:** 2022-02-27

**Authors:** Hairun Liu, Xiaohong Wang

**Affiliations:** a Department of Tissue Engineering, School of Intelligent Medicine, China Medical University (CMU), Shenyang, China; b The Third Department of Orthopeadics, Jinzhou Central Hospital, Jinzhou, China

**Keywords:** ADSCs, osteogenic differentiation, EGFL6 recombinant protein

## Abstract

Adipose-derived mesenchymal stem cells (ADSCs) are a class of pluripotent stem cells isolated from the adipose tissue; they can differentiate into osteoblasts after induction and play an important role in bone repair. EGFL6 protein is secreted by adipocytes and osteoblasts and can promote endothelial cell migration and angiogenesis. This study aimed to explore the effect of recombinant EGFL6 protein on the osteogenic differentiation of ADSCs. The cells were incubated with fluorescein isothiocyanate-conjugated antibodies and analyzed by flow cytometry. Alizarin red staining and alkaline phosphatase staining were used to detect the osteogenic differentiation ability. mRNA expression was analyzed by real-time quantitative polymerase chain reaction (RT-qPCR). Protein expression was determined using Western blotting. The osteogenic differentiation ability of ADSCs isolated from the adipose tissue was significantly weakened after EGFL6 knockdown; this ability was restored upon the addition of EGFL6 recombinant protein. BMP2 knockdown inhibited the effect of EGFL6 recombinant protein on osteogenic differentiation. EGFL6 recombinant protein promoted osteogenic differentiation of ADSCs through the BMP2/SMAD4 signaling pathway. This may provide a potential target for the osteogenic differentiation of ADSCs.

## Graphiical abstract

**Figure:**
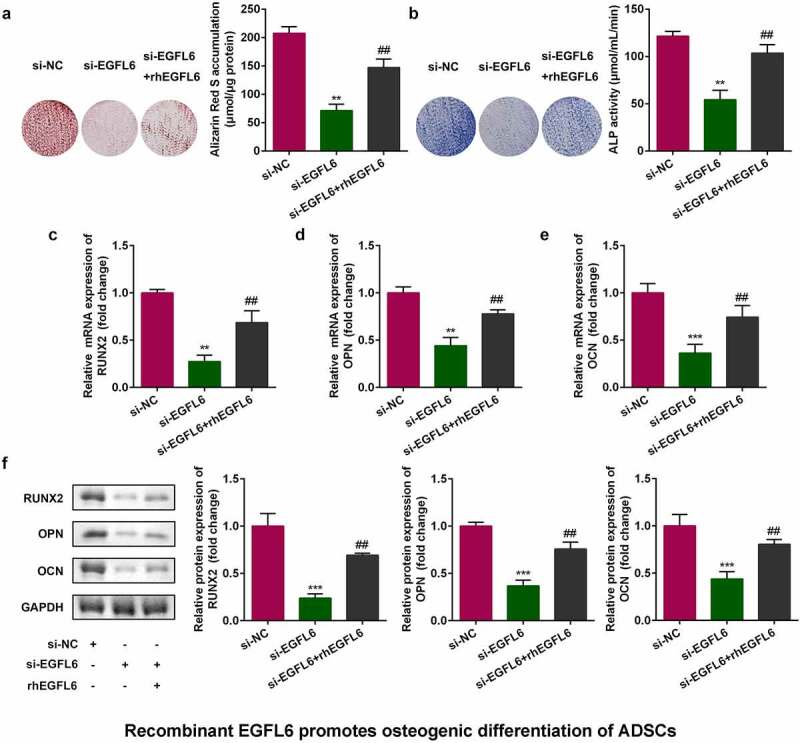

## 1. Introduction

Bone metabolism is a dynamic balanced process in which osteoclasts (OCs) absorb old bone and osteoblasts (OBs) to form new bone [1]. The imbalance between OC and OB induces metabolic bone diseases such as osteoporosis. With the aging population, the incidence of osteoporosis is increasing every year [2]. Additionally, osteoporosis-induced bone fractures reduce the quality of life of the elderly population [3]. Therefore, promotion of bone formation has been the focus of research in recent years. Bone formation is generally regulated by various signaling pathways, including BMP/Smad, PI3K/AKT, Wnt/β-catenin, MAPK, Notch, and Hedgehog [4–7]. Identification of upstream regulatory factors may provide new therapeutic targets for metabolic bone diseases.

Patient-derived cell therapy is an effective method for treating bone-related diseases. Human mesenchymal stem cells (MSCs) are abundant autologous cells that can differentiate into multiple cell lines *in vitro* and *in vivo* and have been recognized as an ideal stem cell source for cell therapy [8,9]. Adipose stem cells (ADSCs) are MSCs present in the adipose tissue and can differentiate into various cells and tissues under specific induction conditions [10]. Experiments have shown that ADSCs have therapeutic effects on various diseases such as skeletal and muscle diseases [11], cardiovascular diseases [12], and autoimmune diseases [13]. ADSCs can differentiate into osteoblasts, lipids, and chondroblasts, similar to classic bone marrow-derived mesenchymal stem cells (BMSCs) [14]. In addition, compared to BMSCs, ADSCs have the characteristics of extensive sources, easy materials, and simple operation [15,16]. Therefore, ADSCs have great potential in the treatment of bone-related diseases.

Epidermal growth factor-like domain protein 6 (EGFL6), located on chromosome Xp22, is a member of the EGF repeat domain superfamily [17]. EGFL6 has been reported to be expressed in early developing adipose tissue, is secreted by adipocytes, and promotes proliferation of adjacent vascular endothelial cells [18,19]. Studies have shown that EGFL6 is abnormally expressed in some tumor tissues [20,21]. In addition, EGFL6 can be secreted by osteoblast-like cells to promote endothelial cell migration and angiogenesis through the ERK signaling pathway [22]. To date, the potential expression and role of EGFL6 in bone biology remain to be elucidated.

Therefore, this study aimed to explore the function and role of EGFL6 in osteogenic differentiation. We hypothesized that EGFL6 promoted the osteogenic differentiation of ADSCs through the BMP2/SMAD4 signaling pathway.

## 2. Materials and methods

### 2.1 Cell culture

The adipose tissues were obtained from seven donors, and ADSCs were isolated according to the modified protocol reported by Zuk et al. [23]. The ADSCs were cultured in osteogenic medium (OM) consisting of proliferation medium, 100 nM dexamethasone (Sigma-Aldrich), 20 μM L-ascorbic acid (Wako Pure Chemical Industries Ltd), 10 mM β-glycerol phosphate (Sigma-Aldrich), and 1% (v/v) penicillin/streptomycin. The cells were maintained in humidity of 95% with 5% CO_2_ at 37°C. ADSCs at passage 3 were selected for further experiments.

The small interfering RNA EGFL6 (si-EGFL6 1# and 2#), si-BMP2, and their negative controls (si-nc) were synthesized by GenePharma (Shanghai, China). All these plasmids were transfected into the cells by using Lipofectamine 2000 (Invitrogen, Carlsbad, USA) for 48 h at 37°C.

### 2.2 Flow cytometry assay

According to a previous study [24], the cells were incubated with fluorescein isothiocyanate-conjugated antibodies (anti-CD90, anti-CD105, anti-CD44, anti-CD14, and anti-CD106) (BioLegend, San Diego, CA, USA) and analyzed using NovoCyte Advanteon B4 Flow Cytometer and NovoSampler Q software (Agilent Technologies Co., Ltd).

### 2.3 Alizarin red staining

According to a previous study [25], ADSCs were cultured in 35-mm plates for 14 days. After fixation in 95% ethanol, the ADSCs were stained with 1% Alizarin red S (ARS) staining solution (Sigma‐Aldrich) at room temperature for 15 min. To quantify the mineralization levels, densitometric analysis of staining was performed using ImageJ 1.48.

### 2.4 Alkaline phosphatase staining and activity

According to a previous study [26], cells were fixed with 4% paraformaldehyde (PFA) for 10 min at room temperature. The cells were then seeded and stained with ALP for 20 min. Images were captured using a scanner. The ALP Assay Kit was used to detect ALP activity according to the manufacturer’s protocol.

### 2.5 RT-qPCR

According to a previous study [27], RNA was extracted from ADSCs using a commercially available kit (Takara, Japan). cDNA was then synthesized, and PCR was carried out using a Real-Time PCR Detection System (Bio-Rad, USA). The sequences of the primers used were as follows: EGFL6: forward: 5′-AAGCTTGGATCCGAATTCAGTATGCAGCCGCCCTGG-3′, reverse: 5′-CTCGAGTCTAGAAGATCTACCTTCTACAGATAAAAAGT-3′; Runx2: forward: 5′-CGCATTCCTCATCCCAGTAT-3′, reverse: 5′-TGTAGGTAAAGGTGGCTGGG-3′; osteocalcin: forward: 5′-GCGCTCTGTCTCTCGTGACCT-3′, reverse, 5′-ATAGATGCGTTTGTAGGCGG-3′; osteopontin: forward: 5′-TCTGATGAGACCGTCACTGC-3′, reverse: 5′-TCTCCTGGCTCTCTTTGGAA-3′; BMP2: forward: 5′-GACTGCGGTCTCCTAAAGGTCG-3′, reverse: 5′- CTGGGGAAGCAGCAACGCTA-3′; SMAD4: forward: 5′- CCATCAGTCTGTCTGCTGCT-3′, reverse: 5′-TGATGCTCTGTCTCGGGTAG-3′; GAPDH: forward: 5′-GAAGGTGAAGGTCGGAGTC-3′, reverse: 5′- GAAGATGGTGATGGGATTTC-3′; and U6: forward: 5′-CTCGCTTCGGCAGCACA-3′, reverse: 5′- AACGCTTCACGAATTTGCGT-3′.

### 2.6 Western blot

According to a previous study [28], protein extracts were loaded onto 10% SDS gel and electrophoresed. The protein extracts were then transferred to the PVDF membrane (Millipore) and incubated with primary antibodies at 4°C overnight. The next day, the membrane was incubated with secondary antibodies for 2 h at room temperature. Finally, the protein bands were captured using ECL system (Thermo Fisher Scientific, Inc.).

### 2.7 Statistical analysis

Statistical analyses were performed using GraphPad Prism 7.0 (Graph-Pad Software, CA, USA). Data have been expressed as means ± standard deviation (SD). Differences between two groups were measured using Student’s *t*-tests. Comparisons between more than two groups were analyzed using analysis of variance. Statistical significance was set at *P* <0.05.

## 3. Results

This study demonstrated that EGFL6 knockdown decreased the red calcium nodules, the activity of blue ALP, and the mRNA levels of osteogenic differentiation-related genes. Recombinant EGFL6 promotes osteogenic differentiation of ADSCs by regulating the BMP2/SMAD4 signaling pathway.

### 3.1 Characterization of ADSCs

**Figure 1.**
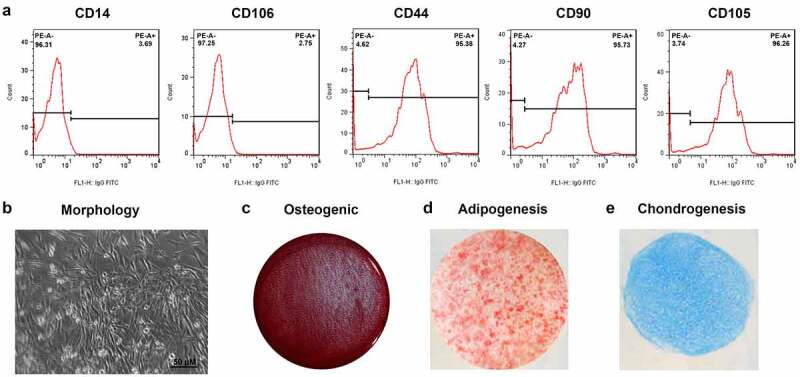
Characterization of ADSCs. (a) Flow cytometry analysis for the detection of ADSC surface markers. (b) Morphology of ADSCs. (c) Osteogenic ADSCs. (d) Adipogenic ADSCs.

To identify the adipocytes, we detected the expression of positive markers CD44 (95.38%), CD90 (95.73%), and CD105 (96.26%), while negative markers CD14 as well as CD106 were 3.69% and 2.75%, respectively (Figure 1(a)). The cells had a typical spindle shape and multidirectional differentiation potential (Figure 1(b)). After induction, the cells could differentiate in osteogenic and adipogenic directions (Figure 1(c,d)).

### 3.2 EGFL6 knockdown attenuates the osteogenic differentiation of ADSCs

**Figure 2.**
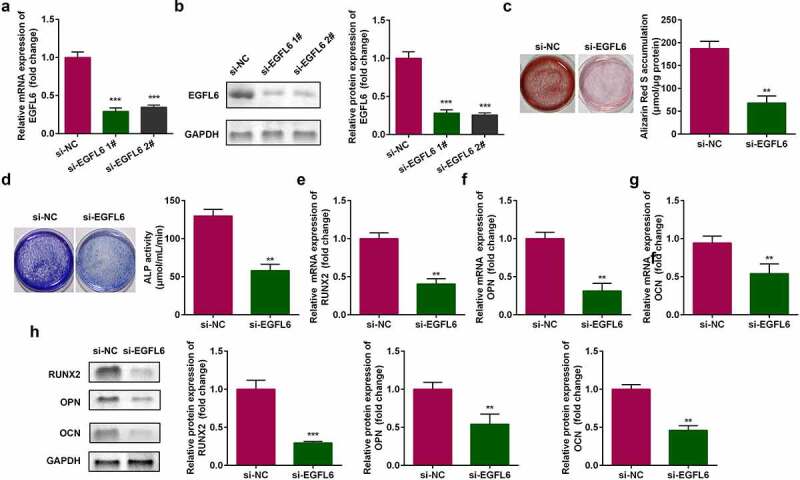
EGFL6 knockdown attenuates the osteogenic differentiation of ADSCs. (a) mRNA expression of *EGFL6* in ADSCs. (b) Protein expression of EGFL6 in ADSCs. (c) ARS staining of ADSCs. (d) ALP activity of ADSCs. (e) mRNA expression of *RUNX2* in ADSCs. (f) mRNA expression of *OPN* in ADSCs. (g) mRNA expression of *OCN* in ADSCs. (h) Protein expression of RUNX2, OPN, and OCN. **P < 0.01, *** *P* < 0.001 versus si-NC.

Compared with si-NC group, the mRNA and protein expression of EGFL6 were significantly decreased after si-EGFL6 1# and si-EGFL6 2# transfection, indicating that the transfection was successful, and si-EGFL6 1# was used in subsequent experiments (Figure 2(a,b)). Cell staining showed that red calcium nodules and the activity of blue ALP notably decreased after EGFL6 knockdown (Figure 2(c,d)). The mRNA levels of osteogenic differentiation-related genes *RNUX2*, osteopontin (*OPN*), and osteocalcin (*OCN*) were remarkably decreased, and the protein expression was also decreased (Figure 2(e-h)).

### 3.3 Recombinant EGFL6 promotes osteogenic differentiation of ADSCs

**Figure 3.**
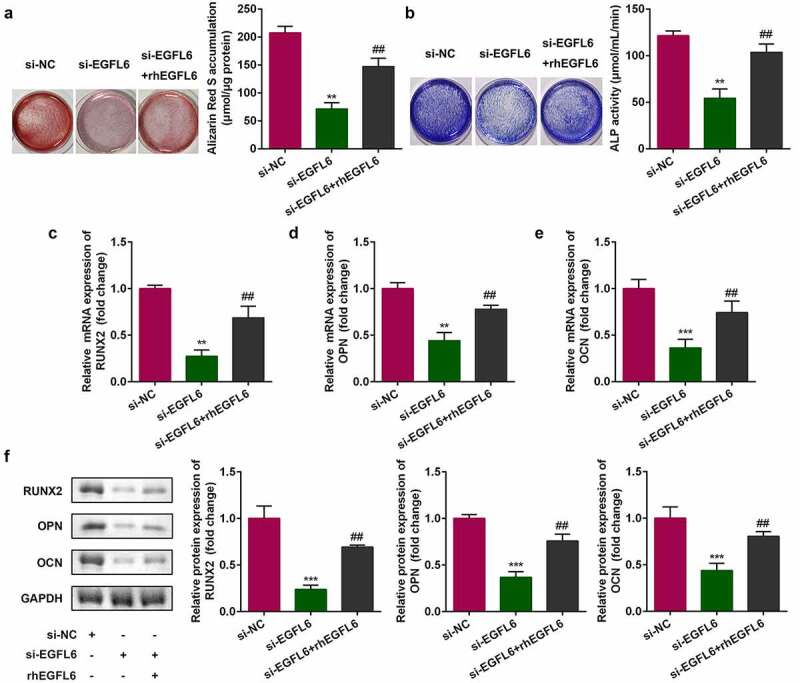
Recombinant EGFL6 promotes osteogenic differentiation of ADSCs. (a) ARS stain of ADSCs. (b) ALP activity of ADSCs. (c) mRNA expression of RUNX2 in ADSCs. (d) mRNA expression of OPN in ADSCs. (e) mRNA expression of OCN in ADSCs. (f) Protein expression of RUNX2, OPN, OCN. ##*P* < 0.01, **P < 0.01, ***P < 0.001 versus si-NC.

Figure 3(a) shows that after EGFL6 knockdown, when the cells were treated with EGFL6 recombinant protein, the red calcium nodules were markedly increased after cell staining, and the activity of blue ALP was also observably enhanced (Figure 3(b)). In addition, the addition of EGFL6 recombinant protein notably increased the mRNA and protein levels of *RNUX2, OPN*, and *OCN* (Figure 3(c-f)).

### 3.4 Recombinant EGFL6 enhances osteogenic differentiation through the BMP2/SMAD4 signaling pathway

**Figure 4.**
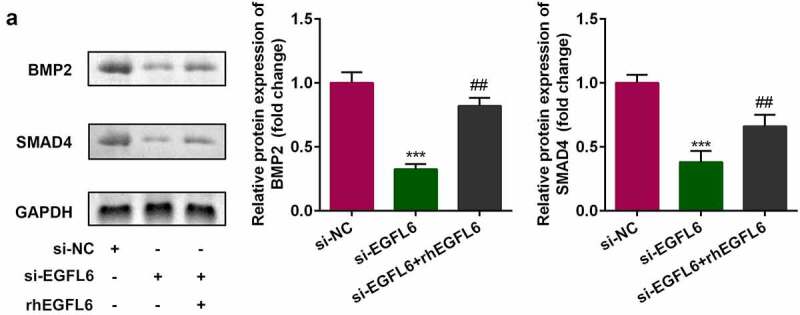
The expression of BMP2 and SMAD4 is positively correlated with EGFL6. (a) Protein expression of BMP2 as well as SMAD4. ##*P* < 0.01, ***P < 0.001 versus si-NC.

**Figure 5.**
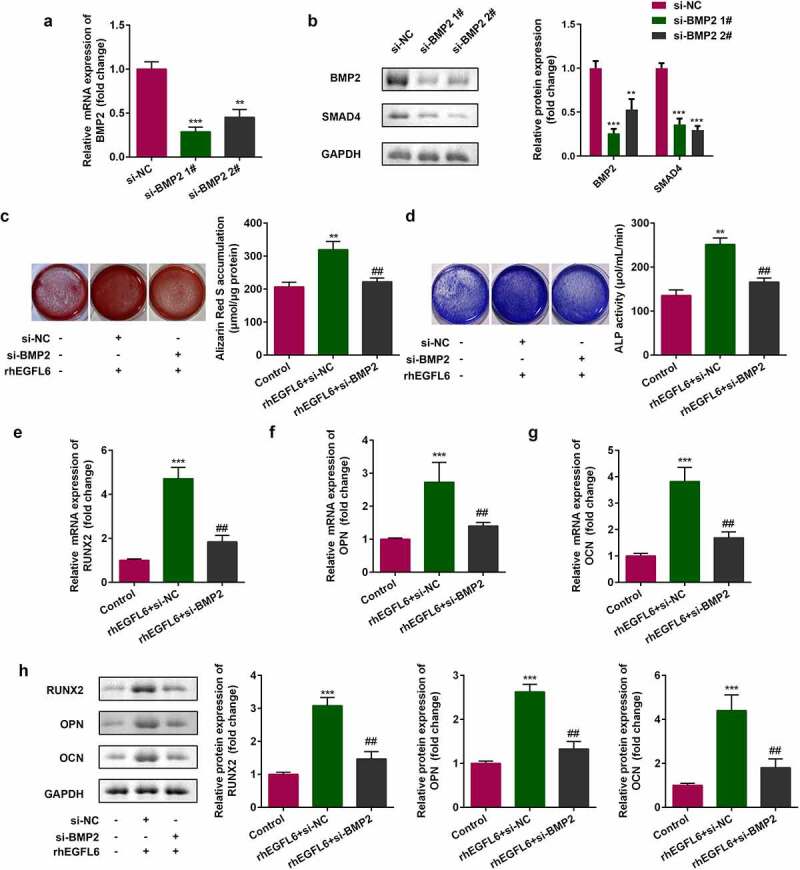
Recombinant EGFL6 enhances osteogenic differentiation through the BMP2/SMAD4 signaling pathway. (a) mRNA expression of *BMP2*. (b) Protein expression of BMP2 and SMAD4. (c) ARS staining of ADSCs. (d) ALP activity of ADSCs. (e) mRNA expression of *RUNX2* in ADSCs. (f) mRNA expression of *OPN* in ADSCs. (g) mRNA expression of *OCN* in ADSCs. (h) Protein expression of RUNX2, OPN, and OCN. ##*P* < 0.01, **P < 0.01, ***P < 0.001 versus si-NC.

In EGFL6 knockdown cells, the expression of BMP2 and SMAD4 decreased, while that of BMP2 and SMAD4 increased noticeably after adding recombinant EGFL6 protein (Figure 4(a)). Compared with the NC group, the mRNA level and protein expression of *BMP2* were significantly decreased after knockdown, and the downstream SMAD4 protein expression was also decreased, indicating that cell transfection was successful. 1# was used for the subsequent experiments (Figure 5(a,b)). Compared with the si-NC group, red calcium nodules and the activity of blue ALP were significantly increased after adding recombinant protein EGFL6, while the effect was inhibited by BMP2 knockdown (Figure 5(c,d)). In addition, the inhibition of BMP2 in cells treated with recombinant EGFL6 markedly reduced the mRNA level and protein expression of *RNUX2, OPN*, and *OCN* (Figure 5(e-h)).

## 4. Discussion

Osteoblasts are mainly differentiated from mesenchymal stem cells and regulate the synthesis, secretion, and mineralization of the bone matrix and osteoclasts [29,30]. Studies have shown that mesenchymal stem cells can be isolated from the adipose tissue and obtained in large quantities [31] and can be induced into osteoblasts, chondrocytes, adipocytes, and other directional transformation [32–34]. In this study, the osteogenic differentiation of ADSCs was weakened after EGFL6 knockdown, while it was restored after the addition of recombinant EGFL6 protein. In addition, the expression of EGFL6 positively regulated BMP2 and SMAD4 proteins, suggesting that EGFL6 promoted the osteogenic differentiation of ADSCs through the BMP2/SMAD4 signaling pathway.

EGFL6 is a secretory protein with structural homology, and the gene is present on human Xp22 chromosome [35]. It has been reported that EGFL6 is involved in the development of a variety of tumors, such as ovarian cancer [36] and colon cancer [37]; however, its role in the regulation of osteogenic differentiation of ADSCs is rarely studied. In this study, the osteogenic differentiation of cells was significantly weakened upon the knockdown of EGFL6 in ADSCs, whereas the ability of osteogenic differentiation was restored after the addition of EGFL6 recombinant protein. These results indicate that EGFL6 plays an important role in the regulation of the osteogenic differentiation of ADSCs.

BMPs are important members of TGF-β superfamily. BMP2, BMP4, BMP7, and BMP9 play important roles in the regulation of osteoblast differentiation [38]. It has been reported that increasing the mRNA expression of *BMP2* can significantly increase the expression of alkaline phosphatase, osteocalcin, Smad, and other osteogenic markers, as well as the formation of mineralized nodules, and the cells are significantly differentiated into osteoblasts [39]. In this study, BMP2 and SMAD4 protein expression was positively correlated with EGFL6, and inhibition of BMP2 expression weakened the promotion of EGFL6 recombinant protein expression on osteogenic differentiation.

In general, EGFL6 recombinant protein can promote the osteogenic differentiation of ADSCs by regulating the BMP2/SMAD4 signaling pathway. Our research provided a novel method and theoretical basis for the clinical treatment of osteoporosis. In the future, we will focus on the clinical application of EGFL6. The role of EGFL6 in ADSCs was further proved by in vivo experiments.

## Highlights

1. EGFL6 knockdown attenuated the osteogenic differentiation of ADSCs.

2. Recombinant EGFL6 promotes osteogenic differentiation of ADSCs.

3. The expression of BMP2 and SMAD4 was positively correlated with EGFL6.
